# Low Tensile Strength Suture With Transosseous Tunnels and Suture Anchors 5 mm in Diameter or Greater Are Associated With Higher Failure Rates in Primary Patellar Tendon Repair

**DOI:** 10.1016/j.asmr.2024.100908

**Published:** 2024-02-14

**Authors:** Mark S. Katsma, Vaughn Land, S. Hunter Renfro, Hunter Culp, George C. Balazs

**Affiliations:** a Bone & Joint Sports Medicine Institute, Naval Medical Center Portsmouth, Portsmouth, Virginia, U.S.A.; b Department of Surgery, Uniformed Services University of the Health Sciences, Bethesda, Maryland, U.S.A.

## Abstract

**Purpose:**

To determine the rate of and risk factors for clinical failure and return to military duty following primary patellar tendon repair with either transosseous trunnel repair or suture anchor repair.

**Methods:**

The Military Health System Data Repository (MDR) was queried to identify all adult patients undergoing surgical treatment of a patellar tendon rupture in the Military Health System from 2014 to 2018. Patients who underwent either transosseous tunnel repair or suture anchor repair were included. Health records were examined to collect additional data. Univariate analysis and multivariate logistic regression models were used to determine independent risk factors for rerupture.

**Results:**

A total of 450 knees in 437 patients were included. Transosseous tunnel repair was the most frequently used technique (314/450, 77%), followed by suture anchor repair (113/450, 25%). Rerupture occurred in 33 knees (7%). There was no difference in rerupture rate between transosseous tunnel repair and suture anchor repair (*P* = .15), and this result persisted within the multivariate logistic regression model. Among transosseous tunnel repairs, use of low tensile strength suture was an independent risk factor for repair failure (odds ratio [OR], 3.4; *P* = .016). Among suture anchor repairs, use of anchors 5.0 mm in diameter or greater (OR, 12.0; *P* = .027) was an independent risk factor for repair failure.

**Conclusions:**

There is no statistically significant difference in failure rate between transosseous tunnel repair and suture anchor repair in primary patellar tendon ruptures. However, the use of low tensile strength suture with transosseous tunnels and the use of suture anchors 5.0 mm in diameter or greater resulted in significantly higher failure rates. These data suggest that use of high tensile strength suture in transosseous tunnel repair and use of suture anchors less than 5.0 mm in diameter in suture anchor repair result in lower failure rate in primary patellar tendon repair.

**Level of Evidence:**

Level III, retrospective cohort study.

Patellar tendon ruptures occur at a rate of 0.68/100,000 per year, predominantly in males.^1^ They occur 6 times more often in active-duty military personnel than the nonmilitary population, at 6/100,000 per year.^2^ Repetitive microtrauma due to strenuous training or sports participation may predispose to tendon rupture during sudden strong contraction of the quadriceps muscle.^3^, ^4^, ^5^, ^6^ Surgical repair is required for optimal patient outcome and is generally performed with either transosseous tunnels or suture anchors.^3^^,^^6^

Historically, repair of patellar tendon injuries was treated with transosseous drill tunnels and reapproximation of the tendon to patellar bone with Mason-Allen, Bunnel, or Krackow sutures.^7^, ^8^, ^9^ The use of suture anchors for extensor mechanism repairs (albeit quadriceps tendon repairs) was proposed by Richards and Barber^10^ in 2002 as an alternative to the transosseous technique, in an effort to initiate early mobilization and increase the strength of repair. In 2007, Capiola and Re^11^ published a 3-anchor technique for patellar tendon repair with high tensile strength suture that has become a technique of choice for many surgeons. Some biomechanical studies have shown decreased gap formation under cyclic loading and increased load to failure with suture anchor repair compared with transosseous tunnel repair.^12^, ^13^, ^14^, ^15^, ^16^ Other biomechanical studies, however, have disputed these findings.^17^, ^18^, ^19^ Nonetheless, suture anchor fixation has grown in popularity, with proponents emphasizing the more limited dissection required, lower risk of disrupting the patellar chondral surface, preservation of the blood supply to the fat pad and patella, and lower risk of iatrogenic patellar fracture.^11^^,^^20^

Despite robust biomechanical data, there are limited clinical studies comparing the 2 repair methods. It remains unclear whether the theoretical and biomechanical advantages of suture anchor repair translate to clinical advantages. It also remains unclear whether variations within each technique (suture type and size, anchor type and size, number of core sutures, etc.) affect repair failure rates.

The purpose of this study was to determine the rate of and risk factors for clinical failure and return to military duty following primary patellar tendon repair with either transosseous trunnel repair or suture anchor repair. We hypothesized a higher rerupture rate with use of transosseous tunnel repair compared with suture anchor repair.

## Methods

The Military Health System (MHS) comprises a network of over 550 health care facilities serving 9.6 million active-duty military personnel, military retirees, and their families. The Military Health System Data Repository (MDR) contains data on all health care encounters for MHS beneficiaries. Following approval from our local institutional review board, the MDR was queried through the Military Analysis and Reporting Tool (M2) for all patients with an encounter for *Current Procedural Terminology* (*CPT*) 27380 or 27381 at a military treatment facility between January 1, 2014, and December 31, 2018. The *CPT* code for 27381 was also included to identify any primary repairs inadvertently coded as allograft reconstructions, in order to maximize the number of identified patients.

Patients were eligible for inclusion if they underwent primary repair of a ruptured patellar tendon over the specified period. Patients were excluded if they underwent a procedure other than primary patellar tendon repair (e.g., revision repair, quadriceps tendon repair, or allograft reconstruction), sustained an open tendon rupture, had polytrauma requiring surgical treatment beyond repair of the patellar tendon, had a pre-existing ipsilateral total knee arthroplasty, were younger than 18 years at the time of surgery, or did not have an available operative report with details of the procedure. We also excluded patients who underwent patellar tendon repair in conjunction with partial takedown and debridement for chronic patellar tendinitis, as well as patients sustaining a distal patellar tendon rupture from the tibial tubercle. In our final analysis, we included only solely transosseous tunnel or suture anchor repairs in order to compare the two.

Included patient records were searched for demographic and injury information, medical and surgical history (to include prior ipsilateral/contralateral tendon rupture and prior patellar tendinitis), intraoperative findings (including repair technique and surgical details), details of surgical construct (type and size of core sutures used, suture anchor diameter and material, number of anchors/tunnels and core sutures), postoperative complications, and clinical findings at the time of final examination. Suture type was divided into high tensile strength suture, composed of braided nonabsorbable material over a coated central core (e.g., Fiberwire [Arthrex], Orthocord [DePuy Synthes], Ultrabraid [Smith & Nephew]) and low tensile strength suture consisting of braided material alone (e.g., Ethibond; Johnson & Johnson). Suture anchor material composition was inconsistently documented and not included in the analysis. Repair failure was defined as need for revision patellar tendon repair or, when reoperation did not occur, documented surgeon assessment that repair had failed. The surgeon’s operative report was used to determine the tear type and location; where this was unavailable, the preoperative magnetic resonance imaging report was used. Military disposition (return to duty vs separation from service for medical reasons) was also recorded for active-duty military personnel. Final clinical assessment of each patient was performed by the attending surgeon as documented in the electronic health record.

### Statistical Analysis

Summary data are reported as counts, percentages, medians, and interquartile ranges (IQRs), as appropriate. Continuous variables were assessed for normality using the Kolmogorov-Smirnov test. Results of this testing showed non-normal distributions for all continuous variables collected, including patient age (*P* < .001), body mass index (BMI) (*P* < .001), time to surgery (*P* < .001), number of suture anchors in patients undergoing suture anchor repair (*P* < .001), number of bone tunnels in patients undergoing transosseous tunnel repair (*P* < .001), and number of core sutures placed (*P* < .001).

For the primary outcome (repair failure), the cohort was analyzed as a whole and as subgroups based on repair technique. For assessment of return to military duty, only active-duty military personnel were included in the analysis.

Univariate associations with collected variables were assessed using Fisher exact testing, χ^2^ testing, or Mann-Whitney *U* testing, as appropriate. Significance was set at *P* < .05. All variables showing an association of *P* < .25 were included in a multivariate logistic regression model to determine independent risk factors for rerupture (whole cohort and subgroups) and separation from military service (active-duty military patients only). Prior to inclusion in the regression model, multicollinearity of candidate variables was assessed by calculating the variable inflation factor (VIF), with values >10 indicative of multicollinearity. Missing data within particular variables were few and excluded from analysis on a case-wise basis. All analysis was performed using IBM SPSS Statistics for Macintosh, Version 27.

## Results

**Fig 1:**
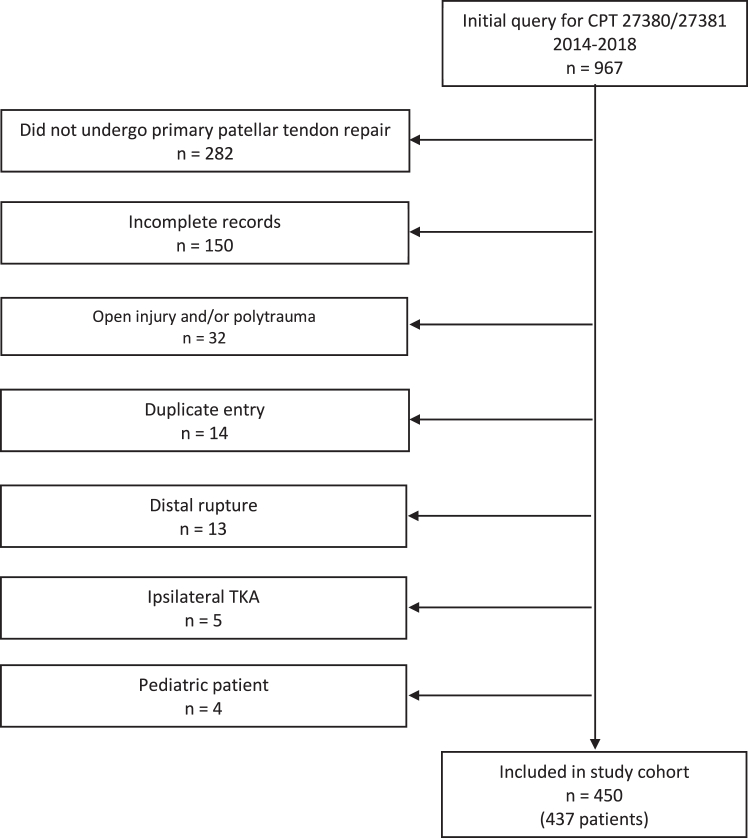
Strengthening the Reporting of Observational Studies in Epidemiology flowchart showing patient inclusions/exclusions.

The initial query yielded 967 knees potentially eligible for inclusion. Following the application of exclusion criteria, a total of 450 knees in 437 patients were included in the final study cohort. See Figure 1 for the Strengthening the Reporting of Observational Studies in Epidemiology diagram of patient selection.

### Demographics

A total of 450 knees in 437 patients (13 patients with bilateral injuries) met inclusion criteria. Median patient age was 35 years (IQR, 14 years). Most patients were male (427/437, 98%), Black race (243/437, 56%), and nonsmokers (342/437, 78%). Median BMI was 28.70 (IQR, 4.95). Median follow-up with orthopaedic surgery was 222 days (IQR, 399 days), and median follow-up with any medical provider in the MHS was 859 days (IQR, 1,607 days). Complete demographic data on the study cohort are given in Table 1.

**Table 1:** Demographics and Potential Risk Factors for Patellar Tendon Rupture in Study Cohort

Characteristic	Value
Age at injury	
Median (IQR), y	35 (14)
Sex, n (%)	
Male	427 (98)
Female	10 (2)
Race, n (%)	
Black	243 (56)
White	124 (28)
Asian	14 (3)
Other/unknown	1 (0)
BMI	
Median (IQR)	28.70 (4.95)
Tobacco use, n (%)	
Smoker	95 (22)
Nonsmoker	342 (78)
Military status, n (%)	
Active duty/guard/reserve	337 (77)
Retiree	61 (14)
Other	39 (8)
History of diabetes/prediabetes, n (%)	
Yes	8 (2)
No	429 (98)
History of thyroid disease, n (%)	
Yes	2 (0)
No	435 (100)
History of contralateral patellar tendon rupture, n (%)	
Yes	35 (8)
No	415 (92)
Pre-existing tendinitis/tendinopathy, n (%)	
Yes	83 (18)
No	367 (82)

BMI, body mass index; IQR, interquartile range.

Most of the cohort (337/437, 77%) were active-duty military personnel. When compared with non-active-duty patients, active-duty military personnel were younger at the time of injury (median age 33 years vs 48 years, *P* < .001), had a lower BMI (median 28.21 vs 30.70, *P* < .001), were less likely to have a diagnosis of diabetes or prediabetes (0.3% vs 7%, *P* < .001), and were less likely to have any nonmusculoskeletal medical comorbidity (27% vs 63%, *P* < .001).

Most patients were injured during recreational sporting activities (76%). Nearly half of patients (40%) were injured playing basketball, with 10% injured playing football. Only 12 patients (3%) were injured during military training activities.

### Intraoperative Findings and Repair Techniques

Most tears were complete inferior pole avulsion injuries (394/450, 88%), with the remainder classified as complete mid-substance ruptures (32/450, 7%) and complex tears with injury at both the patellar bone–tendon attachment and an intratendinous component (24/450, 5%).

Median time to surgery was 6 days postinjury (range, 0-72 days; IQR, 6 days). Most knees underwent transosseous tunnel repair (314/450, 70%), with the remainder undergoing suture anchor repair (113/450, 25%), direct tendon-to-tendon repair (12/450, 3%), or hybrid fixation using both transosseous tunnels and suture anchors (11/450, 2%).

A complete summary of operative techniques and implant utilization is contained in Table 2.

**Table 2:** Operative technique and implant utilization details

Characteristic	Value
Fixation technique (n = 450)	
Bone tunnels	314 (70)
Suture anchors	113 (25)
Direct repair	12 (3)
Hybrid	11 (2)
Bone tunnel fixation (n = 314)	
Days to surgery (n = 314)	3 (8)
Number of bone tunnels (n = 307)	3 (0)
Number of core sutures (n = 310)	4 (0)
Suture vs tape (n = 302)	
Suture	292 (97)
Tape	8 (3)
Combination	2 (1)
Suture type (n = 302)	
High tensile strength	241 (80)
Regular or low tensile strength	61 (20)
Suture size (n = 283)	
#2	155 (55)
#5	128 (45)
Suture anchor fixation (n = 113)	
Days to surgery (n = 113)	4 (7)
Number of suture anchors (n = 113)	2 (0)
Number of core sutures (n = 111)	4 (4)
Suture vs tape (n = 112)	
Suture	90 (80)
Tape	21 (19)
Combination	1 (1)
Suture type (n = 112)	
High tensile strength	108 (96)
Regular or low tensile strength	4 (4)
Suture size (n = 97)	
#1	4 (4)
#2	89 (92)
#5	4 (4)

NOTE. Values are presented as number (%) unless otherwise indicated.

For knees undergoing suture anchor repair, a median of 2 anchors (range, 2-3) and 4 core sutures (range, 4-12) were employed. For knees undergoing transosseous tunnel repair, a median of 3 tunnels (range, 1-4) and 4 core sutures (range, 2-8) were used.

Among knees undergoing suture anchor repair, most used knot-tying suture anchors (78/113, 69%), followed by knotless anchors (33/113, 29%). The remaining 2 knees received a combination of both anchor types. A summary of suture anchor diameter is given in Table 3.

**Table 3:** Diameter of Anchors Used Among Patients Receiving Suture Anchor Repair

Diameter	Repair Failure, n	No Repair Failure, n
<5.0 mm diameter	1	55
2.3 mm	0	1
2.8 mm	0	3
2.9 mm	0	4
3.0 mm	0	1
3.4 mm	0	1
4.5 mm	0	13
4.75 mm	1	32
5.0 mm or greater	11	43
5.0 mm	3	3
5.5 mm	8	40
Mixed sizes∗	0	2
Unknown diameter∗	0	1

∗ Excluded from analysis.

### Clinical Outcomes

Clinical outcomes of the study cohort are summarized in Table 4. At the time of final follow-up, thigh atrophy was documented as present in 161 of 450 (36%) knees. Median flexion at final follow-up was 130 degrees (IQR, 10 degrees). An extension lag was present in 10% (46/450), with a median lag of 5 degrees (range, 2-20 degrees; IQR, 5 degrees). Strength on manual muscle testing was full in 78% (353/450) of knees.

**Table 4:** Outcomes and Complications in the Study Cohort

Characteristic	Value
Quadriceps atrophy at final follow-up	161 (36)
Final flexion ROM (median/IRQ)	130 (10)
Extensor lag at final follow-up	46 (10)
Median lag (IQR)	5 (5)
Strength simplified (final follow-up)	
5/5	353 (78)
4+/5 or less	97 (22)
Complications	62 (11)
Repair failure	33 (7)
Acute event	29 (88)
Attritional	4 (12)
Surgical site infection	10 (2)
VTE (DVT/PE)	4 (1)
DVT	3
PE	1
Dysesthesias/CRPS	2 (0)
Arthrofibrosis	7 (2)
Patella fracture	2 (0)
Reoperation	48 (11)
I&D(s)	6 (13)
Revision repair	17 (35)
Revision reconstruction	9 (19)
MUA ± scope LOA	5 (10)
Multiple	4 (8)
Distalizing TTO with cartilage repair	1 (2)
Knee scope loose body removal	4 (8)
Patella ORIF	1 (2)
ACLR	1 (2)

NOTE. Values are presented as number (%) unless otherwise indicated.

ACLR, anterior cruciate ligament reconstruction; CRPS, complex regional pain syndrome; DVT, deep vein thrombosis; I&D, irrigation and debridement; IQR, interquartile range; LOA, lysis of adhesions; MUA, manipulation under anesthesia; ORIF, open reduction internal fixation; PE, pulmonary embolism; ROM, range of motion; TTO, tibial tubercle osteotomy; VTE, venous thromboembolism.

### Complications

The overall complication rate from surgery was 14% (62/450). The most common complication of surgery was repair failure, which occurred in 7% of knees (33/450). Of these, 29 (88%) were associated with a reported acute traumatic event. Three patients developed a postoperative deep vein thrombosis, and 1 developed a postoperative pulmonary embolism. There were 10 surgical site infections (2%).

A total of 48 patients (11%) underwent reoperation during the postoperative period. Revision repair or extensor mechanism reconstruction was performed in 26 patients; the remainder of patients with documented repair failure declined further operative intervention. Five patients (10%) required manipulation under anesthesia with or without arthroscopic lysis of adhesions for arthrofibrosis. On univariate testing, the risk of experiencing any complication following surgery was not significantly associated with age, BMI, sex, race, smoking status, medical comorbidities, tear type, or repair technique.

### Primary Outcome—Repair Failure

Univariate analysis identified no demographic, injury, surgical, or postoperative factor as significantly associated with repair failure. When directly comparing transosseous tunnel repair to suture anchor repair (tendon-to-tendon and hybrid repair excluded), there was no difference in the likelihood of repair failure (6% vs 11%, *P* = .15), but this factor did meet the criterion for inclusion in the regression model. Other nonsignificant associations that met the threshold for inclusion in the logistic regression model were increased BMI (*P* = .22), smoking (*P* = .19), and presence of diabetes/prediabetes (*P* = .11). On multivariate logistic regression modeling, no factor was found to significantly predict repair failure.

#### Subgroup Analysis—Transosseous Tunnel Repair

Among patients who underwent transosseous tunnel repair, univariate analysis identified no significant association with failure any factor. Four variables met the threshold for inclusion in the multivariate logistic regression model: smoking (*P* = .16), tear location (*P* = .14), number of core sutures (*P* = .24), and type of core suture (*P* = .06).

Within the logistic regression model, use of low tensile suture was 3.4 times more likely to result in repair failure when compared with high tensile suture (13% vs 5%; odds ratio [OR], 3.4; *P* = .016).

#### Subgroup Analysis—Suture Anchor Repair

Among patients who underwent suture anchor repair, univariate analysis showed that an increasing number of core sutures were significantly associated with a higher risk of repair failure (median 8 core sutures in failed cases vs 4 sutures in nonfailed cases, *P* = .01). There was also a statistically significant association between repair failure and the use of anchors 5.0 mm in diameter or greater (20% failure rate with the use of 5.0 mm diameter anchors or greater vs 2% failure rate with the use of anchors less than 5.0 mm in diameter, *P* = .002). Three additional variables with nonsignificant association met the threshold for inclusion in the logistic regression model. These included younger age (*P* = .22), higher BMI (*P* = .17), and diabetes (*P* = .11).

Within the logistic regression model, only the use of suture anchors 5.0 mm in diameter or greater independently predicted repair failure with suture anchor repair (OR, 12; *P* = .027).

### Return to Military Duty

Of the 337 active-duty military personnel in the cohort, 300 (89%) were able to return to military duty, and 37 (11%) required separation from service for medical reasons.

On univariate analysis, there was a significant association between the need for medical separation and failure of primary repair (*P* = .009), the presence of any postoperative complication (*P* = .018), the presence of documented muscle atrophy at final follow-up (*P* = .05), decreased knee flexion at final follow-up (*P* = .037), and less than full side-to-side quadriceps strength at final follow-up (*P* < .001).

Seven additional variables did not reach statistical significance on univariate analysis but met the threshold for inclusion in a multivariate logistic regression model. These were younger patient age (*P* = .1), smoking (*P* = .21), branch of military service (*P* = .052), history of contralateral patellar tendon rupture (*P* = .056), fixation technique (*P* = .23), reoperation for any reason (*P* = .16), and the presence of an extensor lag at final follow-up (*P* = .08). No multicollinearity was present between reoperation (VIF 4.9), repair failure (VIF 5.1), and the presence of any complication (VIF 2.2).

Within the multivariate logistic regression model, only the presence of less than full quadriceps strength at final follow-up predicted the need for separation from military service for medical reasons (OR, 3.2; *P* = .008).

## Discussion

The most important finding of this study is that there is no significant difference in failure rate of primary patellar tendon repair using transosseous tunnel repair versus suture anchor repair. However, there is a 3.4 times increased risk of repair failure with transosseous tunnel repair when low tensile strength suture (vs high tensile strength suture) is used, and there is a 12 times increased risk of failure with suture anchor repair when anchors 5.0 mm in diameter or larger are used.

Our findings are similar to an older cohort reported on by Fredericks et al.,^2^ who used the MDR to review 504 patellar tendon repairs performed between 2010 and 2015 in only active-duty military personnel. In their cohort, transosseous tunnel repair was used in 81% of cases, suture anchor repair was performed in 7%, and the method of repair could not be determined in 12%. In our series, 25% of patients underwent suture anchor repair, likely reflecting the increased popularity over time of suture anchor repair. While neither study found a significant difference in failure rate between the 2 techniques, we did identify a somewhat higher overall repair failure rate at 7%, versus 3% in Fredericks et al.^2^ While the reasons for this change are unclear, it may reflect our inclusion of military retirees and dependents, who are typically older with more medical comorbidities. Additionally, Fredericks et al.^2^ did not perform subgroup analysis of the individual repair techniques, and so it is unclear whether our findings on the influence of suture type and anchor size were similar in their cohort.

O’Dowd et al.^21^ compared 321 transosseous tunnel repairs with 53 suture anchor repairs in a nonmilitary cohort, finding a significantly higher failure rate with the transosseous technique. They also reported that suture anchor repair had an upward trend in use toward the end of their 2008-2015 collection period. O’Dowd et al.^21^ reported no failures with suture anchor repair, but the type and size of anchors employed were not reported.^21^ Unlike our cohort, all transosseous tunnel repairs were performed with high tensile strength suture, whereas 20% of the transosseous tunnel repairs in this series used low tensile strength braided nonabsorbable suture. Their reported failure rate with transosseous tunnel repair was 7.5%, which is similar to the 5% failure rate for the technique in our series when high tensile strength suture was used. Low tensile strength suture demonstrated a 3.4 increased odds of failure in transosseous bone tunnel repairs (*P* = .016).^21^ High tensile strength nonabsorbable suture is made up of a polyethylene core suture with a myriad of different coatings to improve handling and strength (Fiberwire, Orthocord, Maxbraid, Ultrabraid), whereas low tensile strength suture is a braided polyester. High tensile has been shown to perform with better knot security, load to failure, and stiffness and strain properties, which may explain our findings.

In general, studies examining clinical outcomes of extensor mechanism repair treat all repair constructs within a particular technique as equivalent. While this approach simplifies analysis, it may also obscure important differences in outcomes using different materials and implant types. Our finding that the largest diameter anchors result in the highest risk of repair failure may be explained by the larger diameter holes required, decreasing the (already small) available surface area for tendon-to-bone healing. This, in turn, may create a weaker healed bone-tendon unit. We were unable to locate any basic science studies clearly answering whether repaired tendons heal over the site of anchor insertion, but the results of this study do raise the question of whether further basic science research in this area is warranted.

### Limitations

The primary limitation of this study is its retrospective nature, whereby the accuracy of the study data is wholly dependent on accurate documentation in the medical records reviewed, potentially underestimating comorbidities and other injury factors. The median follow-up of 7 months may have been in sufficient for all patients to reach a point of maximum medical improvement, and further follow-up may have potentially resulted in lower rates of extension lag and persisted weakness. Additionally, a total of 150 potentially eligible patients were excluded from his study due to lack of available operative notes and/or complete preoperative/postoperative documentation, which also could have affected our findings. Our findings are also limited by the large number of surgeons contributing patients to this cohort. We did not examine whether failure rates varied between individual surgeons and facilities. While we attempted to gather granular data on the suture and implants used in this study cohort, certain data were not examined that could have influenced outcomes, such as manufacturer and implant design/suture anchor material. Overall, the short follow-up bias and the data heterogeneity potentially confound the results we have obtained. Finally, it is possible that we were underpowered to detect significant differences for certain subgroup analyses. For example, using the observed failure rates, a sample size of 628 transosseous repairs and 226 suture anchor repairs would be the minimum number to show a difference between groups. Achieving such large sample sizes is challenging in studies where extremely granular data are gathered from medical records manually, and few large clinical databases contain the degree of detail analyzed in this study.

## Conclusions

There is no statistically significant difference in failure rate between transosseous tunnel repair and suture anchor repair in primary patellar tendon ruptures. However, the use of low tensile strength suture with transosseous tunnels and the use of suture anchors 5.0 mm in diameter or greater resulted in significantly higher failure rates. These data suggest that use of high tensile strength suture in transosseous tunnel repair and use of suture anchors less than 5.0 mm in diameter in suture anchor repair result in lower failure rate in primary patellar tendon repair.

## Disclosure

The authors report the following potential conflicts of interest or sources of funding: G.C.B. has received educational payments from Fortis Surgical and hospitality payments from 10.13039/100008894Stryker, outside the submitted work, and is Associate Editor of *Arthroscopy Journal*. All other authors (M.S.K., V.L., S.H.R., H.C.) declare that they have no known competing financial interests or personal relationships that could have appeared to influence the work reported in this paper*.* Full ICMJE author disclosure forms are available for this article online, as supplementary material.

## Supplementary Data

ICMJE author disclosure forms
